# Functional and Structural Correlates of Impaired Enrichment-Mediated Adult Hippocampal Neurogenesis in a Mouse Model of Prenatal Alcohol Exposure

**DOI:** 10.3233/BPL-200112

**Published:** 2020-12-29

**Authors:** Kymberly Gustus, Lu Li, Jessie Newville, Lee Anna Cunningham

**Affiliations:** Department of Neurosciences, University of New Mexico School of Medicine, Albuquerque, NM, USA

**Keywords:** Pattern discrimination learning, dentate gyrus, immediate-early genes, fetal alcohol spectrum disorders

## Abstract

**Background:**

Fetal alcohol spectrum disorders (FASDs) are associated with a wide range of cognitive deficiencies.

**Objective:**

We previously found that gestational exposure to moderate levels of alcohol in mice throughout the 1st-2nd human trimester-equivalents for brain development results in profound impairment of the hippocampal neurogenic response to enriched environment (EE) in adulthood, without altering baseline neurogenesis rate under standard housing (SH). However, the functional and structural consequences of impaired EE-mediated neurogenesis in the context of prenatal alcohol exposure (PAE) have not been determined.

**Results:**

Here, we demonstrate that PAE-EE mice display impaired performance on a neurogenesis-dependent pattern discrimination task, broadened behavioral activation of the dentate gyrus, as assessed by expression of the immediate early gene, c-Fos, and impaired dendritic branching of adult-generated dentate granule cells (aDGCs).

**Conclusions:**

These studies further underscore the impact of moderate gestational alcohol exposure on adult hippocampal plasticity and support adult hippocampal neurogenesis as a potential therapeutic target to remediate certain neurological outcomes in FASD.

## INTRODUCTION

Fetal alcohol spectrum disorders (FASD) are the leading preventable cause of intellectual and neurodevelopmental disability, and impose considerable financial and economic burden on societies [1]. The prevalence of FASD among school-age children within the USA has been estimated at ∼2.5% [2]. A recent CDC report indicates that approximately 1 in 10 women consume alcohol at some point during pregnancy [3], yet few interventions are available for mitigating the cognitive and neurobehavioral disabilities associated with FASD [4]. Evidence from clinical and preclinical FASD research has shown that gestational exposure to even moderate levels of alcohol can result in impairment of brain function [5]. Although prenatal alcohol exposure (PAE) affects many brain regions, structural and functional impairments of the hippocampus are particularly well documented [6–9].

Adult hippocampal neurogenesis represents a unique form of neural plasticity that promotes flexible learning and adaptive behavioral responses to cognitive and emotional challenge [10–15], which are aspects of mental function often disrupted in FASD [16–19]. Our laboratory first reported a negative impact of moderate PAE on adult hippocampal neurogenesis in mice 20]. Using both continuous and limited access voluntary drinking paradigms to model moderate PAE (maternal blood alcohol concentrations ∼80–120 mg/dl), and genetic labeling methods to identify the neurogenic lineage, we demonstrated that although gestational exposure to moderate levels of alcohol had no effect on adult neurogenesis under standard housing conditions, it resulted in severe impairment of the neurogenic response to enriched environment (EE) [20–22]. These findings suggest that even moderate gestational alcohol exposure might also impair the ability of FASD individuals to respond optimally to human correlates of EE, or to respond optimally to behavioral training/interventions known to improve hippocampal-associated behaviors in humans [23, 24]. This is relevant given that moderate alcohol consumption is the most common pattern of consumption among women who report drinking alcohol during pregnancy [25].

Taken together, these observations suggest that restoring EE-mediated postnatal hippocampal neurogenesis may improve certain neurological outcomes associated with moderate gestational alcohol exposure. Validation of this hypothesis, however, requires the ability to pinpoint functional consequences of impaired EE-mediated neurogenesis within the context of PAE, since PAE impairs many other aspects of hippocampal plasticity. In the current study, we sought to elucidate the behavioral consequences of impaired EE-mediated neurogenesis in PAE mice using a neurogenesis-dependent A-B contextual discrimination task, and to determine whether impairment of EE-mediated neurogenesis in PAE mice is associated with alterations in hippocampal network activation and/or dendritic maturation of adult-generated DGCs (aDGCs), which could mediate these behavioral deficits.

## MATERIALS AND METHODS

### Mice

C57Bl6/J or Nestin-CreER^T2^: tdTomato bitransgenic mice were used for these studies. C57Bl6/J mice were purchased from Jackson Laboratories. Nestin-CreER^T2^: tdTomato bitransgenic mice were obtained from breeding colonies maintained in the University of New Mexico Health Sciences Center Animal Facility. For transgenic mice, breeding colonies were maintained for homozygosity at both the Nestin-CreER^T2^ [26] and Ai9 (RCL-tdT) transgene loci [27], on a C57BL/6J genetic background as previously described [21, 28]. Genotyping was routinely performed by PCR analysis of tail genomic DNA as previously described in detail [28]. All mice were housed under reverse 12-hr dark/12-hr light cycle (lights off at 08:00 h) in a humidity and temperature controlled room with food and water available *ad libitum*. Animal procedures were approved by the University of New Mexico Institutional Animal Care and Use Committee in accordance with NIH policies on Humane Care and Use of Laboratory Animals.

### Prenatal alcohol exposure (PAE)

PAE offspring were generated within the New Mexico Alcohol Research Center Scientific Core, using a well-characterized limited-access “drinking-in-the-dark” gestational ethanol exposure paradigm as previously described in detail [29]. Food and water consumption, maternal weight gain, litter size, pup weight, pup retrieval times and time on nest does not differ between the alcohol-exposed and control animals in this drinking paradigm [29]. Briefly, 60 day old C57Bl6/J or Nestin-CreER^T2^:tdTomato female mice were offered a 0.066% saccharin solution containing EtOH, instead of water, for 4 hrs per day from 10:00–14:00. (note: C57Bl/6J mice were used for c-Fos experiments, whereas Nestin-CreER^T2^:tdTomato transgenic mice were used for all other experiments). Following a 5 day gradual ramp-up period (from 0–10% EtOH), the mice were subsequently maintained on a 10% EtOH drinking regimen for 2 weeks prior to pregnancy and throughout gestation. Female mice offered 0.066% saccharin (SAC) without EtOH during the same periods served as controls. Consumption volume during the 4 hr access period, as determined for dams beginning after one week of drinking 10% alcohol, was 5.74 ± 0.41 gm/kg, *n* = 22 (range 3.46 – 10.90 gm/kg; median 4.82 gm/kg). In this paradigm, blood alcohol concentrations directly correlate to the average amount of ethanol consumed over the 4 hr drinking period throughout gestation [29]. Based on this correlation, average daily maternal blood alcohol concentrations (BACs) were estimated to be 80–90 mg/dL throughout gestation.

### Tamoxifen administration

Tamoxifen (TAM; Sigma-Aldrich, St. Louis, MO, USA) was dissolved in 10% EtOH/90% sunflower oil (Sigma-Aldrich) and administered by intraperitoneal injections (180 mg/kg) to Nestin-CreER^T2^:tdTomato bitransgenic mice (SAC and PAE offspring) for five consecutive days for quantification of aDGCs as previously described [21, 22, 28] or as a single injection (180 mg/kg) for dendritic morphological analysis at the ages indicated in text.

### Standard housing (SH) and Enriched environment (EE)

PAE and SAC offspring were gender segregated at weaning and housed thereafter in standard housing (SH) or enriched environment (EE) conditions until sacrifice. SH comprised a standard mouse cage (28 cm×18 cm×13 cm) without running wheels or toys (three mice/cage). EE included a larger cage (48 cm×27 cm×20 cm) containing two running wheels and multiple objects (ladder, tunnel and hanging toys that were changed out weekly; six mice/cage) as previously described [20, 22].

### A-B Contextual-fear discrimination

**Fig. 1:**
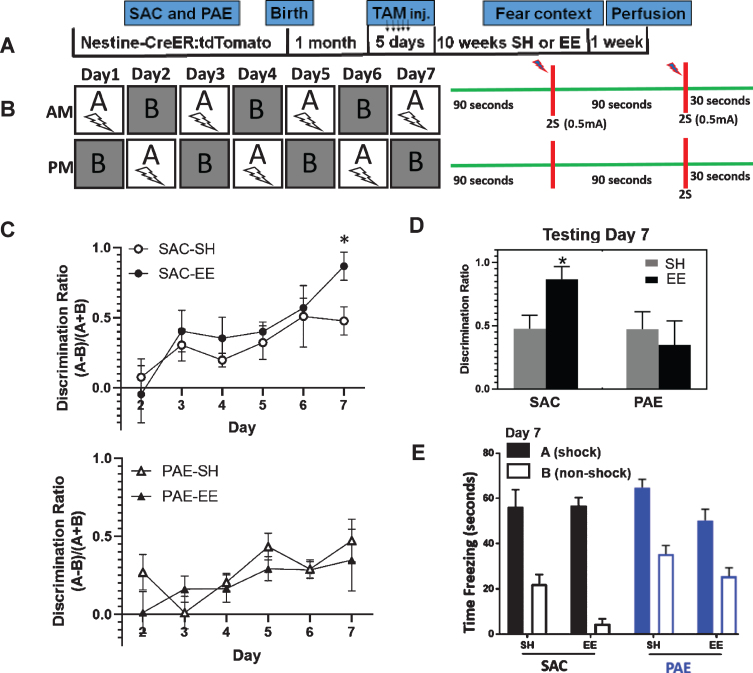
PAE impairs EE-mediated improvement A-B context fear discrimination learning. A. Experimental timeline. SAC and PAE Nestin-CreERT2:tdTomato mice received tamoxifen dosing to induce reporter expression in aDGCs and were subjected to SH or EE housing conditions for 10 weeks prior to behavioral testing. B. Experimental design for the A-B context fear discrimination task. Mice received two 2-second 0.5 mA foot shocks separated by a 90 second interval in Context A only. The first shock occurred 90 seconds following placement into the chamber and mice were removed from the chamber 30 seconds following the 2^nd^ foot shock. In the non-shock context B, mice received no foot shock or other aversive event. Each mouse experienced one daily session in Context A and one daily session in Context B, separated by 3 hours. The order of context exposure was alternated each day for 7 days. Daily discrimination scores were calculated as: (freezing time context A - freezing time context B) / (freezing time context A + freezing time context B) during the first 90 seconds of each testing session. C. Discrimination learning over time. Data are plotted as the mean daily discrimination scores per group±SEM. Group n’s were as follows: SAC-SH (*n* = 8 mice) and SAC-EE (*n* = 8 mice) sampled across 5 separate litters; PAE-SH (*n* = 15 mice) and PAE-EE (*n* = 15 mice) sampled across 7-8 separate litters. Three way ANOVA statistics: testing day [F(6,250) = 325, *p* < 0.0001], alcohol treatment [F(1,250) = 175, *p* < 0.0001], housing [F(1,250) = 12.49, *p* = 0.0005)], alcohol treatment x housing [F(1,250) = 98.73, *p* < 0.0001], alcohol treatment x housing x day [F(6,250) = 18.38, *p* < 0.0001]. *p < 0.01 SAC-EE vs. all other groups (Tukey’s multiple comparison). D. Mean discrimination scores at testing day 7. *p < 0.01 SAC-EE vs. all other groups (Tukey’s *post-hoc* comparison). E. Freeze time in Context A (shock) and Context B (non-shock) across all experimental groups on testing day 7. Data expressed as mean±SEM.

Behavioral testing was performed in the University of New Mexico Center for Brain Recovery and Repair Preclinical Core Facility using a protocol blinded to treatment. Mice were trained on the A-B contextual-fear discrimination learning task as previously described [22, 30, 31], and originally modified from Sahay et al., [32] (Fig. 1A and 1B). This behavioral assay tests the cognitive ability of mice to learn to discriminate between two similar contexts, using a protocol that is dependent upon the activity of aDGCs [24, 31–33]. All experiments were performed using the automated NIR Video Fear Conditioning System for Mouse (Med Associates, Fairfax, VT). Briefly, Context A (shock condition) was comprised of a standard chamber with a stainless steel floor with steel grid rods arranged in a straight horizontal plane, clear Plexiglas front wall, and aluminum side and back walls. A similar chamber comprised Context B (non-shock), except for a non-shock floor comprised of stainless steel grid rods arranged in 2 offset horizontal planes, and side and back walls covered in striped black and white contact paper. All mice were habituated in a red-light holding room for one hour before the first training session and were returned to their home cages in the animal facility following the final training session each day. Each mouse was individually placed into either Context A (foot shock) or Context B (no foot shock) for 90 s, followed immediately by foot shock (0.5 mA) for 2 s in Context A only. Following a 90 s interval, mice in Context A received a second foot shock and were removed from the chamber 30 s later. Mice in Context B received no shock or other aversive stimulus during the trial and remained within the chamber for the same period of time. Three hours following the first session, mice were exposed to a second identical training session, except that the mouse initially subjected to Context A (shock) was subjected to Context B (no shock), and *vice versa*. This sequence was repeated once per day for 7 consecutive days, with the order of testing in Context A *vs.* Context B reversed every day for each individual mouse. Freezing time was measured with an automated software system (Video Fear Conditioning “Video Freeze^®^” Software, Med Associate, Fairfax, VT) for analysis of digitally-recorded motion (30 frames per second) under red-light conditions. Freezing was defined using a motion threshold of 10 and minimum freeze duration of 30 frames (i.e., subject must remain immobile for one second to be registered as a freeze). A discrimination score was automatically calculated for each mouse for each day of training [(freezing score Context A - freezing score Context B)/(freezing score Context A + freezing score Context B)], during the first 90 s within either chamber. Thus, higher discrimination scores indicate better contextual discrimination. All mice were returned to home cages (EE or SH) and sacrificed 1 week later (∼11 weeks post-tamoxifen and ∼4.0 months of age) and brains from 4 mice per experimental group were randomly selected for histological quantification of tdTomato^+^ aDGCs as described below.

### Exposure to novel environment

Mice were transferred in their home cage to a nearby behavioral testing room and acclimated for one hour before exposure to novel environment. Each mouse was individually removed from its home cage and placed into an open field novel environment for 30 minutes, followed by return to home cage for 80 minutes prior to sacrifice for c-Fos expression analysis. All experiments were performed under infrared lighting conditions during the reversed light/dark cycle. Control mice not exposed to novel environment remained within their home cages in their home room undisturbed until sacrifice.

### Immunohistochemistry

All mice were overdosed with sodium pentobarbital (150 mg/kg, Fort Dodge Animal Health, Fort Dodge, IA) administered by intraperitoneal injection, and transcardially perfused with phosphate-buffered saline (PBS) containing 0.1% procaine and 2 U/ml heparin, followed by 4% paraformaldehyde (w/v) in 0.1 M PBS. The brains were post-fixed overnight, cryoprotected with 30% sucrose (w/v) in 0.1 M PBS for 48 hours at 4°C and sectioned in the coronal plane at 40*μ*m (quantification of cell numbers) or 100*μ*m (dendritic morphology analysis) as indicated in text, using a freezing sliding knife mictrotome. Free-floating tissue sections were immunostained using primary antibodies directed against the neuronal nuclear antigen, NeuN (1:1000, EMD Millipore, MAB 377) or against the immediate early gene, c-Fos (1:1000, Calbiochem, PC-38T) and visualized using FITC-conjugated or Cy3-conjugated secondary antibodies (1:250; Jackson Immunoresearch Laboratories, Westgrove, PA), respectively. Where indicated, sections were counterstained with 4’,6-diamidino-2-phenylindole (DAPI, Thermo Fisher Scientific, Carlsbad, CA). All sections were mounted onto glass slides and cover slipped using Fluoromount G mounting media (Electron Microscopy Sciences, Hatfield, PA). Z-stack maximum projection images were obtained using LASX acquisition software linked to a Leica DMi8 TCS SP8 confocal microscope (Wetzlar, Germany) using a 20X oil immersion objective. Images were subsequently tiled and stitched using LASX or Adobe Photoshop Software (Adobe Systems Inc., San Jose, CA) to generate representative photomicrographs.

### Quantification of tdTomato^+^/NeuN^+^ aDGCs and c-Fos^+^ nuclei

The number of tdTomato^+^/NeuN^+^ co-labelled aDGCs or c-Fos^+^ nuclei were quantified using the Optical Fractionator probe in StereoInvestigator software (MicroBrightfield Biosciences, Williston, VT) linked to an Olympus IX-51 DSU spinning disk confocal microscope using a 40X objective as previously described [28]. For comparing tdTomato^+^/NeuN^+^ aDGCs across groups, the number of co-labeled aDGCs within the dentate gyrus of the dorsal hippocampus were estimated across two coronal sections per mouse. Regions of interest (ROI) were manually outlined using a 10X objective and followed the outer limits of the granule cell layer and 1 cell thickness into the hilus. Two sections spaced 480*μ*m apart between –1.28 and –2.12 mm relative to bregma were quantified per mouse using an exhaustive counting scheme with 150×150*μ*m grid size. The numbers of c-Fos positive nuclei within the suprapyramidal blade of the dorsal dentate gyrus were similarly estimated utilizing three coronal sections (between –1.28 and –2.12 to bregma) spaced 240*μ*m apart and an identical exhaustive counting scheme. The optical disector height was set at 15*μ*m with 2*μ*m top and bottom guard zones.

### Dendritic morphological analysis

For dendritic morphological analysis, PAE and SAC NestinCreER^T2^:tdTomato mice received a single dose of tamoxifen (180 mg/kg, intraperitoneal) at 47–51 days of age and were maintained in EE housing conditions until sacrifice at 6 weeks post-tamoxifen (∼3.5 months of age at sacrifice). Mice were sacrificed by sodium pentobarbital followed by transcardial perfusion with 4% paraformaldehyde, with post-fixation and tissue cryoprotection as described above. Brains were sectioned in the coronal plane at 60–100*μ*m thickness, mounted on glass slides and coverslipped with Fluoromount G as described above. Z-stack images were obtained using the Leica DMi8 TCS SP8 confocal microscope and LASX acquisition software. A 20× oil immersion objective (1*μ*m optical sections) was used to capture the dendritic tree and a 63× oil immersion objective (zoom 5, and z-step size of 0.15*μ*m) was used to capture spine images. Z stacks of dendrites were subsequently processed into maximum projection 2D images for further analysis. Spine images were processed using Huygens Deconvolution (Scientific Volume Imaging, Hilversum, Netherlands) prior to 3D reconstruction using Imaris^TM^ image analysis software (Bitplane, Concord, MA).

Dendrites from individual tdTomato^+^ aDGCs were traced using 2D Neurolucida software (MBF Bioscience, Williston, VT) and analyzed for branching complexity (Sholl analysis), total dendritic length and cell body area using Neurolucida Explorer. Dendritic protrusions (spines) were imaged from segments 20–75*μ*m in length (40*μ*m average) located within the middle 1/3 of the dendritic tree of tdTomato^+^ aDGCs located within the suprapyramidal blade of the dentate gyrus. Dendritic segments were digitally reconstructed in 3D utilizing Imaris Filament Tracer and Spine Detection Software (Bitplane, Concord, MA), and subjected to automated spine quantification and classification using Imaris Classify Spines Xtension. Dendritic protrusions were binned into the following categories using default measurement rules that are based on morphological maturity (Toni et al., 2007; Berry and Navadi, 2017): Stubby spines (<1*μ*m in length), long thin spines (protrusion > 3*μ*m in length with head width > neck width), mushroom spines (protrusion length < 3*μ*m and a head width > 2× neck width) and filopodia (remaining dendritic protrusions). Two to five tdTomato^+^ aDGCs were sampled and averaged for each mouse for Sholl analysis (n/group = mouse number/group), and 3 dendritic sections were sampled per mouse for spine analysis (n/group = mouse number/group), with 4–6 mice per group across 2–6 distinct litters as indicated in figure legends.

### Statistical Analysis

This study was restricted to male offspring for consistency due to limited availability of mice for some experiments. Future work will focus on characterization of female mice, given that prior research has demonstrated sex differences in vulnerability to alcohol toxicity [34, 35].

All data were analyzed using ANOVA with appropriate *post-hoc* analyses as indicated in text using GraphPad Prism 8.3.0 (La Jolla, CA). Data are expressed as means±SEM, with *p*-values < 0.05 considered significant.

## RESULTS

### Impaired EE-mediated improvement in discriminative learning in PAE mice is directly correlated with impaired neurogenesis

The A-B contextual-fear discrimination behavioral assay tests the cognitive ability of mice to learn to discriminate between two similar contexts [24, 36] and requires the activity of adult-generated DGCs (aDGCs) for optimal performance [30–32, 37–39]. Here, we tested whether impairment of EE-mediated neurogenesis in PAE mice is directly correlated with impaired performance on this behavioral task. Figures 1A and 1B depict the experimental timeline and 7 day behavioral testing protocol, respectively. Briefly, SAC and PAE NestinCreER^T2^:tdTomato male offspring received daily tamoxifen injections for five consecutive days at one week post-weaning to induce tdTomato reporter gene expression in hippocampal nestin^+^ stem/progenitor cells and their downstream progeny [21, 22, 28, 30, 31], and were subsequently placed into standard housing (SH) or enriched environment (EE) for 10 weeks. We then tested all mice on a neurogenesis-dependent version of the A-B context-fear discrimination learning task, where mice learn to discriminate the shock context (context A) from a similar but distinct non-shock context (context B). Discrimination was quantified using an automated scoring method based on freezing behavior as previously described [22, 30, 31]. We began testing all mice at 10 weeks post-tamoxifen, as prior work demonstrated that PAE-EE mice display ∼50% fewer aDGCs compared to SAC-EE mice at this time point [20–22].

Although all mice learned the discrimination task over time, there were significant differences across groups. Analysis of the discrimination ratios by three-way repeated measures ANOVA revealed significant discrimination learning across all groups over time (testing day F(6,250) = 325, *p* < 0.0001, Fig. 1C). Significant main effects of alcohol treatment [F(1,250) = 175, *p* < 0.0001] and housing [F(1,250) = 12.49, *p* = 0.0005)] were detected, with significant interactions of treatment x housing [F(1,250) = 98.73, *p* < 0.0001] and treatment x housing x day [F(6,250) = 18.38, *p* < 0.0001]. As shown in Fig. 1D, by testing day 7 SAC-EE mice displayed an approximate 2-fold increase in discrimination ability compared to their SAC-SH controls (*p* < 0.0001; Tukey’s post-hoc multiple comparison). In contrast, discrimination scores of PAE-EE mice were no different from PAE-SH mice at day 7. Figure 1E depicts average freezing times in context A and context B across all groups on testing day 7, demonstrating that the improved discrimination scores were due to reduced freezing time in the non-shock context, with SAC-EE mice showing greatest improvement compared to all other groups. These data demonstrate that EE-mediated improvement in a neurogenesis-dependent version of the A-B contextual fear discrimination task is impaired in PAE mice.

**Fig. 2:**
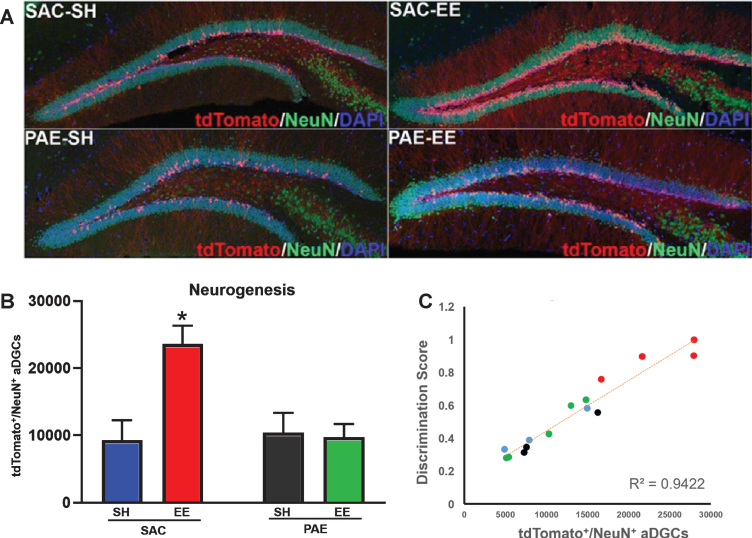
Impaired EE-mediated discrimination learning in PAE mice is directly correlated with impaired neurogenesis. A. Representative confocal microscopy images of coronal sections through the dorsal dentate gyrus demonstrating tdTomato^+^ aDGCs (red), NeuN^+^ mature postmitotic neurons (green), DAPI^+^ nuclear counterstain (blue). B. Number of tdTomato+/NeuN+aDGCs across groups (means±SEM). Two-way ANOVA statistics: alcohol treatment [F (1,11) = 5.83, *p* = 0.03)], housing [F(1,11) = 6.74, *p* = 0.02], alcohol treatment x housing interaction [F(1,11) = 8.07, *p* = 0.01]. *p = 0.02 SAC-SH vs. SAC-EE; *p* < 0.01 SAC-EE vs. PAE-EE (Tukey’s *post-hoc* analysis). N = 4 mice/group. C. Behavioral performance as a function of neurogenesis. Colored circles correspond to data from individual mice from SAC-SH (blue), SAC-EE (red), PAE-SH (black) and PAE-EE (green); i.e., color convention as in B. Pearson correlation, R^2^ = 0.9422.

To assess the relationship between behavioral performance and adult hippocampal neurogenesis, we sacrificed all mice within one week of the final behavioral testing day (mice age ∼4 months) and quantified the number of tdTomato^+^ aDGCs within the dorsal dentate gyrus from 4 randomly selected mice per group. As shown in Fig. 2A, tdTomato^+^/NeuN^+^ aDGCs were robustly labeled across all groups, with an obvious increase in the number of aDGCs in SAC-EE mice. As shown in Fig. 2B, SAC-EE mice displayed a > 2-fold increase in the number of tdTomato^+^ aDGCs compared to SAC-SH controls (*p* = 0.02), whereas PAE-EE mice displayed no change in the number of tdTomato^+^ aDGCs compared to PAE-SH mice, consistent with previous reports [20–22]. Two-way ANOVA of tdTomato^+^ aDGC cell counts revealed main effects of alcohol treatment [F (1,11) = 5.83, *p* = 0.03)], housing [F(1,11) = 6.74, *p* = 0.02] and a significant treatment x housing interaction [F(1,11) = 8.07, *p* = 0.01]. Importantly, the number of aDGCs across individual mice was directly correlated with the level of performance on the neurogenesis-dependent A-B contextual fear-discrimination task at training day 7 (R^2^ = 0.9422), such that mice with the highest number of aDGCs displayed the highest discrimination scores and *vice versa* (Fig. 2C).

### Increased c-Fos expression within the dentate gyrus granule cell layer of PAE-EE mice following exposure to novel environment

**Fig. 3:**
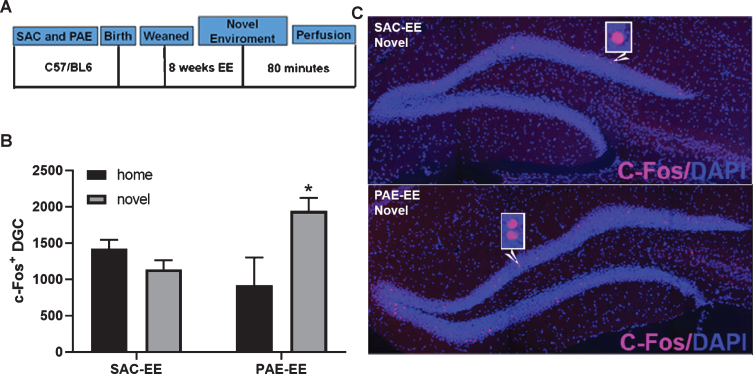
PAE-EE mice display broadened novelty-induced c-Fos expression within the dentate gyrus compared to SAC-EE mice. A. Experimental timeline. SAC and PAE C57Bl6/J mice were exposed to EE housing conditions for 8 weeks (SAC-EE and PAE-EE). Mice were then exposed to a novel environment for 30 minutes and sacrificed 80 minutes following return to home cage. SAC-EE and PAE-EE mice not exposed to novelty remained in home cage until sacrifice and served as controls. B. c-Fos immunofluorescence. Representative confocal images of c-Fos immunoreactivity (pink) within dorsal dentate gyrus in SAC-EE and PAE-EE mice following exposure to novel environment. Histological sections were counterstained with DAPI nuclear dye (blue). C. Quantification of c-Fos^+^ nuclei within the suprapyramidal blade of the dorsal dentate gyrus across groups (mean±SEM). Two-way ANOVA statistics: alcohol treatment x novelty interaction [[F (1,21) = 10.03, *p* = 0.005]. Tukey’s *post-hoc* analysis revealed a significant impact of novelty on c-Fos+expression only in PAE-EE mice (*p = 0.01). Group n’s are as follows: SAC-EE home cage (*n* = 5 mice across 5 litters) and SAC-EE novelty (*n* = 7 mice across 7 litters); PAE-EE home cage (*n* = 5 mice across 5 litters) and PAE-EE novelty (*n* = 8 mice across 8 litters).

Recent work suggests that young aDGC contribute to pattern discrimination by facilitating sparse activation within the dentate granule cell layer via local feedback inhibition and/or via direct monosynaptic input onto mature DGCs [40, 41]. To determine whether impaired EE-mediated neurogenesis in PAE mice is associated with alterations in behavioral activation of pre-existing, mature DGCs, we compared expression of the immediate early gene, c-Fos, within the dentate granule cell layer of SAC-EE and PAE-EE mice following exposure to novel environment. For these experiments, we exposed SAC-EE and PAE-EE mice (both groups reared under EE for 8 weeks) to a novel open field environment for 30 minutes and sacrificed them 80 minutes after return to their home cage (Fig. 3A). Control mice were not exposed to the novel environment and remained undisturbed within their home cage until sacrifice. We then quantified the number of c-Fos^+^ nuclei within the superior blade of the dentate granule cell layer of all mice. As shown in Fig. 3C, c-Fos^+^ cells were sparsely distributed throughout the dentate granule cell layer in both groups exposed to novelty; however, PAE-EE mice displayed a > 2-fold increase in the mean number of c-Fos^+^ DGCs compared to SAC-EE mice (Fig. 3B). Two-way ANOVA revealed a significant alcohol treatment x novelty interaction [[F (1,21) = 10.03, *p* = 0.005] with a significant impact of novelty on c-Fos^+^ expression only in PAE-EE mice (*p* = 0.01, PAE-EE home cage *vs.* PAE-EE novelty, Tukey’s *post-hoc* analysis).

### PAE impairs EE-mediated structural maturation of aDGCs

**Fig. 4:**
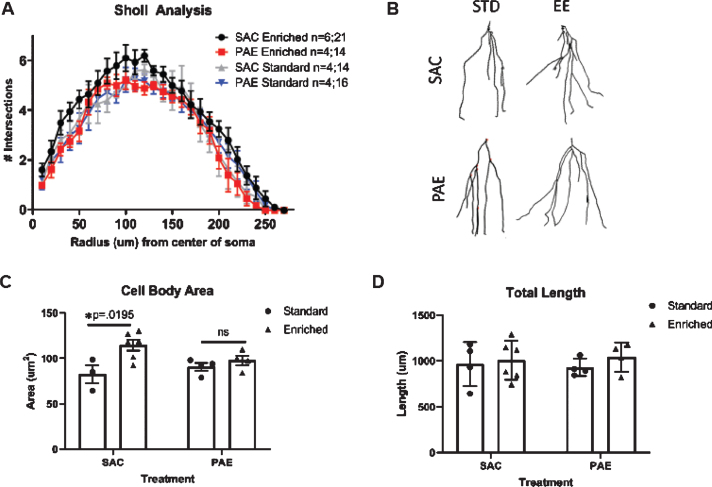
PAE impairs EE-mediated dendritic branching in aDGCs. A. Sholl analysis of dendritic branching complexity in tdTomato^+^ aDGCs across experimental groups. Three way ANOVA statistics: alcohol treatment [F(1,216) = 12.27, *p* < 0.0006], housing [F(1,162) = 9.40, *p* < 0.0025], alcohol treatment x housing interaction [(F(1,162) = 24.70), *p* < 0.0001]. n’s were as follows: SAC-SH (*n* = 4 mice; 14 neurons sampled) and SAC-EE (*n* = 6 mice; 21 neurons sampled) across 6 separate litters; PAE-SH (*n* = 4 mice; 16 neurons sampled) and PAE-EE (*n* = 4 mice; 14 neurons sampled) across 2 separate litters. 2-5 tdTomato^+^ cells were sampled/mouse and averaged such that *n* = mouse constitutes the unit of statistical determination. B. Representative Neurolucida^tm^ traces from compressed Z-stack confocal images for each group. C. Quantification of cell soma size of tdTomato+aDGCs across groups. Mean±SEM *p < 0.02 Tukey’s *post-hoc*. D. Quantification of total dendritic length in tdTomato^+^ aDGCs across groups. Mean±SEM.

EE and spatial learning enhance dendritic complexity of aDGCs in mice [42–48]. Here, we examined the impact of PAE on EE-mediated dendritic branching complexity, using Sholl analysis and computer-assisted reconstruction of dendritic spines. For these studies, Nestin-CreER^T2^:tdTomato reporter mice received a single injection of tamoxifen at approximately 3 months of age to sparsely label aDGCs. Such sparse labeling facilitates dendritic tracing by minimizing dendritic tree overlap among labeled aDGCs. All mice were sacrificed at 6 weeks post-labeling (mice ∼4.5 months of age at sacrifice), dendritic trees were traced and their morphology assessed by computer assisted Sholl analysis. As shown in Fig. 4A, dendritic branching was highest in SAC-EE mice compared to all other groups. Three-way ANOVA of dendritic branching complexity revealed a significant effect of distance from cell soma [F(26, 216) = 53.90, *p* < 0.0001), alcohol treatment [F(1,216) = 12.27, *p* < 0.0006] and housing [F(1,162) = 9.40, *p* < 0.0025] on dendritic branching, with a significant alcohol treatment x housing interaction [(F(1,162) = 24.70), *p* < 0.0001], but no change in total dendritic length (Fig. 4D). Two-way ANOVA comparison of total intersections across groups revealed a significant alcohol treatment x housing interaction [F(1,14) = 6.05, *p* = 0.027] with SAC-EE>PAE-EE (*p* = 0.016, Sidak’s multiple comparison). Representative traces of aDGC dendrites in 2D are shown in Fig. 1B. The increased dendritic complexity in SAC-EE mice was also accompanied by a significant increase in cell body size compared to SAC-SH controls, which was not observed in PAE-EE mice (Fig. 4C).

**Fig. 5:**
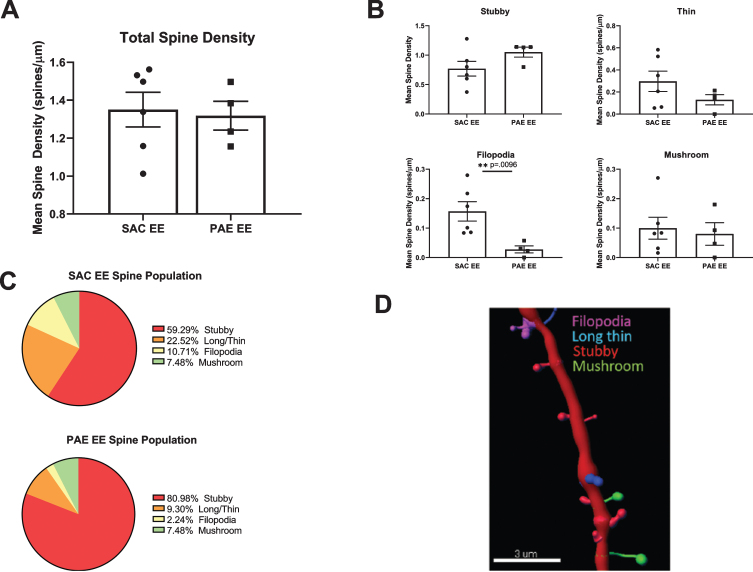
Comparison of spine density and morphology in tdTomato^+^ aDGCs from SAC-EE vs. PAE-EE mice. A. Total dendritic spine densities in SAC-EE vs. PAE-EE mice. SAC-EE (*n* = 6 mice from 5 litters), PAE-EE (*n* = 4 mice from 2 litters). Mean #spines/*μ*m±SEM. B. Densities of stubby, long thin, filopodial and mushroom protrusions in SAC-EE vs. PAE-EE mice. Classification are based on criteria as outlined in Methods. Mean #spines/*μ*m±SEM. *p < 0.01 unpaired *t*-test with Welch’s correction. C. Mean percent distribution of spine classes in tdTomato+aDGCs from SAC-EE and PAE-EE mice. D. Representative 3D reconstruction of dendritic segment with filopodial, long thin, stubby and mushroom spines. Image reconstructed with Imaris^tm^ Filament Tracer software.

To determine whether impairment of EE-mediated dendritic branching in aDGCs from PAE mice was associated with alterations in dendritic spine density or morphology, we sampled 20–75*μ*m dendritic segments (average 40*μ*m) located within the middle third of the dendritic tree from randomly selected SAC-EE and PAE-EE tdTomato^+^ aDGCs. We digitally reconstructed dendritic segments and subjected the reconstructions to automated quantification and classification of dendritic protrusions based on pre-determined morphological criteria as outlined in Methods and depicted in Fig. 5D. Total spine density of 6 week old aDGCs did not differ significantly between SAC-EE and PAE-EE mice (Fig. 5A). However, classification of spine type by morphology revealed 50% fewer filopodia-like spines in 6 week old aDGCs from PAE-EE compared to SAC-EE mice (Fig. 5B). When calculated as percentage of total spines (Fig. 5C), the reduced number of filopodial protrusions was accompanied by an increase in percentage of mushroom spines in PAE-EE (4.14% filopodial, 10.17% mushroom) compared to SAC-EE mice (8.44% filopodial, 8.77% mushroom); however, this increased percentage of mushroom spines did not reach statistical significance (Chi-square statistical analysis), and there were also no significant differences in the distribution of other spine types.

## DISCUSSION

We found that PAE impairs EE-mediated improvement of discrimination learning as assessed using the A-B contextual fear discrimination task, and that impaired learning is directly correlated with impaired EE-mediated neurogenesis across individual mice. Previous work has demonstrated that immature aDGCs uniquely impact hippocampal network activity and are critical for pattern discrimination learning [10]. In the A-B contextual fear discrimination task, the mouse acquires a discrimination over training days, such that fear responses gradually become less in the non-shock compared to the shock context. Suppression of adult neurogenesis, optogenetic silencing of adult generated DGCs, or interference with their plasticity increases generalization between fear and neutral contexts and impairs discrimination learning of this task over time [49]. The ability to discriminate similar episodic memories with overlapping features, such as required for the A-B contextual fear discrimination and other pattern separation tasks depends upon functional adult hippocampal neurogenesis [11, 14, 32, 39]. Sahay et al. originally demonstrated that enhancing neurogenesis using a gain of function genetic approach was sufficient to improve learning on the A-B context fear discrimination task [32] and we previously demonstrated that genetic silencing of aDGCs without impairing their survival markedly impaired learning in this behavioral paradigm [31]. The current studies extend prior work demonstrating impaired A-B context discrimination learning in PAE-EE vs. SAC-EE mice [22], by also comparing learning across SH conditions and by demonstrating a direct correlation between the rate of neurogenesis and behavioral performance on an individual mouse basis. Our observation that PAE-SH mice display similar levels of discrimination learning compared to SAC-SH controls by testing day 7 suggests that, at least in this behavioral test of discrimination learning, non-neurogenic mechanisms related to general disruption(s) of hippocampal function in PAE do not mask neurogenesis-dependent performance.

The mechanisms by which aDGCs promote pattern discrimination learning are not entirely understood, but are thought to involve engagement of inhibitory circuits that facilitate sparse encoding of contextual and spatial information by the dentate granule cell layer [10, 40, 41, 50]. Young aDGCs are less constrained by GABAergic inhibitory input [51–56], are more easily recruited by cortical inputs [57, 58] and exert greater recruitment of GABAergic inhibitory interneurons [59]. Impaired neurogenesis is thereby thought to result in reduced inhibition of mature dentate granule cells, which in turn leads to overlapping patterns of neuronal activation in response to similar but non-identical stimuli [10, 32, 40, 41, 50]. Inhibition of mature, pre-existing DGCs by young aDGCs not only occurs via indirect disynaptic feedback that involves favored recruitment of GABAergic inhibitory interneurons [40] but can also occur via direct monosynaptic innervation of mature DGCs [41]. In the latter case, presynaptic glutamate released by aDGCs stimulates extrasynaptic group II metabotropic glutamate receptors (mGluRII) coupled to G-protein inwardly rectifying potassium channels (GIRKs) expressed on mature DGCs [41]. This occurs primarily in response to activation of aDGCs by the lateral entorhinal cortex(LEC) /lateral performant path (LPP), which is particularly important in transmitting novel information [41, 60, 61]. LPP inputs to the dentate gyrus are most pronounced in the suprapyramidal blade [41, 62] and aDGCs receive greater innervation from the LEC than from medial entorhinal cortex [63, 64]. Consistent with a role for aDGCs in these inhibitory processes, we observed an ∼ 2-fold increase in the number of novelty-activated dentate granule cells within the suprapyramidal blade in PAE-EE compared to SAC-EE mice based on expression of the immediate early gene, c-Fos, a surrogate marker for neuronal activation at the population level. This result was anticipated, given that there are ∼50% fewer aDGCs in PAE-EE compared to SAC-EE mice. This level of disinhibition of mature dentate granule cells is also comparable to that reported in other studies in which adult neurogenesis has been arrested [40, 41]. It should be noted that recent studies have identified a rare and spatially localized subtype of developmentally-generated DGC that dominates granule cell recruitment preferentially within the suprapyramidal blade in response to a range of novelty experiences, but whether these are regulated by young aDGCs has not been investigated [65].

In contrast to PAE-EE mice, SAC-EE mice displayed no increase in c-Fos activation within the dentate gyrus in response to novelty. This is also consistent with prior studies demonstrating that normal mice exposed to EE for prolonged periods habituate faster to novelty compared to SH mice [66, 67]. Stimulation of adult-born granule cells *in vivo* lowers the number of c-Fos compared with controls during novel arena exploration [40]. Taken together, these observations indicate that PAE mice do not appropriately adapt to enriched environment, and strongly suggest that impaired EE-mediated neurogenesis has important consequences for behavioral activation of inhibitory hippocampal circuits thought to mediate both novelty and pattern discrimination learning.

In addition to asking how impaired EE-mediated neurogenesis in PAE mice impacts circuit activation and behavioral output, it is also important to understand whether surviving aDGCs in PAE-EE mice display compensatory mechanisms in an attempt to meet increased hippocampal-engaged cognitive demand with fewer aDGCs. We previously demonstrated that aDGCs from PAE-EE mice display a marked increase in the frequency of spontaneous excitatory postsynaptic currents compared to aDGCs in SAC-EE mice [68]. This suggests a compensatory increase in monosynaptic excitatory afferent input, perhaps in an attempt to meet increased cognitive demand with 50% fewer aDGCs in PAE-EE compared to SAC-EE mice. Prior work has demonstrated that engagement of hippocampal activity and conditions of EE promote dendritic complexity and increased monosynaptic afferent input [42–44, 46, 47]. Surprisingly, we observed a significant impairment of EE-mediated dendritic branching of aDGCs in PAE mice and also an impairment of EE-mediated neuronal maturation as assessed by cell body size, indicating reduced EE-mediated structural plasticity of aDGCs within PAE mice.

Although spine densities were similar in SAC-EE and PAE-EE mice, filopodial protrusions were decreased by approximately 50% in PAE-EE mice, and this was accompanied by an increase in the density of mature mushroom spines that did not reach statistical significance. Filopodia are actin-rich protrusions that emerge from dendrites during neuronal maturation to form contacts with nearby axons [69]. Upon synaptic contact, filopodia transform into thin or mushroom spines with functional synapses or may be pruned if they do not receive inputs [70]. Previous work has demonstrated that both EE and direct high frequency stimulation of performant path inputs to aDGCs increase spine size without changing total spine density [42, 71]. Since our studies were powered to detect >30% differences in spine type, it is difficult to determine whether the decreased number of filopodial protrusions in PAE-EE mice might represent filopodia pruning due to reduced synaptic input vs. ongoing transition of filopodial to more mature spine types that did not reach statistical significance at this time point. This would be most parsimonious with our prior electrophysiological findings of increased spontaneous excitatory synaptic activity in aDGCs from PAE-EE vs. SAC-EE mice, since mushroom spines contain the highest number of postsynaptic glutamatergic receptors [69], thereby providing a substrate for increased afferent excitatory synaptic contact. On the other hand, our findings of impaired EE-mediated dendritic branching and cell body size together with fewer filopodia also supports the former possibility (i.e., reduced synaptic input). It is important to note that in the current study, we assessed dendritic branching and spines in 6 week old aDGCs that experienced 6 weeks of EE, whereas the electrophysiological studies were performed in 8 week old aDGCs exposed to 8 weeks of EE. Thus these different maturational time points could also account for the disparity. An additional caveat includes the region of dendrite analysis, which was restricted to the middle third of the dendritic tree in the current study. This may be relevant, since prior studies have demonstrated heterotopic/homeostatic remodeling of spines along the dendritic tree of aDGCs, depending on source of evoked excitatory activity [71]. Nevertheless, our observations of a 50% reduction of filopodial protrusions in PAE-EE mice warrants further investigation to determine the extent, time course and functional consequences of changes in spine morphologies in PAE-EE vs. SAC-EE mice.

We have previously demonstrated that impaired EE-mediated neurogenesis in PAE mice is largely due to impaired integration of newly generated post-mitotic neurons, and not impaired progenitor proliferation, reduced pool of early progenitors or decreased exploratory activity within the complex enriched environment [20–22, 68]. Potential mechanisms for impaired EE-mediated adult neurogenesis may include altered electrophysiological function of the existing hippocampal circuitry and/or inability of newly generated aDGCs to respond appropriately to activity-dependent integration. Activity-dependent synaptic integration into a functional hippocampal circuit is essential for long-term survival of early post-mitotic aDGCs [72]. Neurogenesis is tightly linked to excitation [73, 74], and EE-mediated neurogenesis requires activation of existing DGCs [75]. Newborn aDGCs require activity-dependent synaptic input for survival during a critical period in their maturation when they display heightened plasticity and lowered threshold for long-term potentiation (LTP) [52], dependent upon appropriate NMDA receptor subunit expression [38]. EE also exerts profound effects on afferent connectivity, including a topographical redistribution of monosynaptic afferent input onto newborn aDGCs that may be important for EE-mediated survival [42]. Thus, disruption of existing circuitry or perturbation of NMDA receptor subunit expression in newly-generated aDGCs in PAE mice could impair activity-dependent survival. PAE is known to result in decreased dentate LTP [76–79] and decreased expression of NMDA receptor subunits [80]. We previously demonstrated impairment of EE-mediated dendritic branching in pre-existing mature DGCs in PAE mice that was associated with blunting of an EE-mediated shift in the excitatory/inhibitory balance of spontaneous synaptic input [68], further indicating that the inappropriate response of the existing circuitry to EE may underlie impaired EE-mediated neurogenesis.

Prior studies utilizing various gestational exposure paradigms have demonstrated that higher doses of alcohol administered throughout gestation or during the early postnatal period in rodents can cause a reduction in baseline neurogenesis, which can be restored back to baseline by EE or exercise [81–83]. However, our studies focus on a moderate gestational alcohol exposure paradigm, in which baseline neurogenesis is unaffected under standard housing conditions, but mice are unable to elicit a neurogenic response to EE above baseline. This effect is dependent upon timing of gestational exposure and does not occur when ethanol exposure is restricted to the first 2 weeks of postnatal life [28]. The relevance of this work for clinical FASD is underscored by mounting evidence for continuation of hippocampal neurogenesis throughout life in humans [84–87] [but see [88]], and the role of neurogenesis in hippocampal-dependent cognitive and behavioral deficits that are also disrupted in FASD [16–19, 89]. Reductions in hippocampal volume and impaired temporal lobe network function that correlate with hippocampal-related behavioral deficits in FASD are also consistent with the possibility of impaired neurogenic function [7, 90–93].

One limitation of the current study is that only males were included. While a complete analysis of sex-dependent effects was beyond the scope of the current study, we previously found no significant sex-dependent effects of PAE on EE-mediated neurogenesis, although the overall level of EE-mediated neurogenesis in SAC-EE mice was slightly greater in females than males [20]. On the other hand, there is evidence that prenatal alcohol can exert sex-dependent effects on structural and functional outcomes [94, 95], and sex-dependent effects on adult neurogenesis after learning have also been reported [96]. Thus, it will be important in future studies to determine whether the behavioral and structural correlates of impaired EE-mediated neurogenesis described here also occur in female PAE mice.

In conclusion, our studies support behavioral relevance for impaired EE-mediated neurogenesis in PAE mice, and provide an additional basis for testing therapeutic strategies that might restore EE-mediated plasticity in our preclinical FASD mouse model of moderate gestation alcohol exposure. The implication is that restoring the neurogenic response to cognitive demand may be required for optimal responsiveness to behavioral training/interventions that target hippocampal-associated behaviors in clinical FASD [23].

## FUNDING INFORMATION

This research was directly supported by the following grants: NIH-NIAAA P50-AA022534; NIH-NIAAA F31AA027127; NIH-NIAAA 1R01AA027462-01A1; NIH-NIGMS 1P20GM109089.

## CONFLICT OF INTEREST

The authors have no conflicts of interest to report.

## References

[1] Greenmyer JR , Popova S , Klug MG , Burd L . Fetal alcohol spectrum disorder: a systematic review of the cost of and savings from prevention in the United States and Canada. Addiction. 2020;115(3):409–17. 31628757 10.1111/add.14841

[2] May PA , Baete A , Russo J , Elliott AJ , Blankenship J , Kalberg WO , et al. Prevalence and characteristics of fetal alcohol spectrum disorders. Pediatrics. 2014;134(5):855–66. 25349310 10.1542/peds.2013-3319 PMC4210790

[3] Tan CH , Denny CH , Cheal NE , Sniezek JE , Kanny D . Alcohol use and binge drinking among women of childbearing age - United States, 2011–2013. MMWR Morbidity and mortality weekly report. 2015;64(37):1042–6. 26401713 10.15585/mmwr.mm6437a3

[4] Kodituwakku PW , Kodituwakku EL . From research to practice: an integrative framework for the development of interventions for children with fetal alcohol spectrum disorders. Neuropsychology Review. 2011;21(2):204–23. 21544706 10.1007/s11065-011-9170-1

[5] Petrelli B , Weinberg J , Hicks GG . Effects of Prenatal Alcohol Exposure (PAE): Insights into FASD using PAE Mouse Models. Biochem Cell Biol. 2018. 10.1139/bcb-2017-0280 PMC5991836 29370535

[6] Autti-Ramo I , Autti T , Korkman M , Kettunen S , Salonen O , Valanne L . MRI findings in children with school problems who had been exposed prenatally to alcohol. Dev Med Child Neurol. 2002;44(2):98–106. 11848116 10.1017/s0012162201001748

[7] Berman RF , Hannigan JH . Effects of prenatal alcohol exposure on the hippocampus: spatial behavior, electrophysiology, and neuroanatomy. Hippocampus. 2000;10(1):94–110. 10706221 10.1002/(SICI)1098-1063(2000)10:1<94::AID-HIPO11>3.0.CO;2-T

[8] Nardelli A , Lebel C , Rasmussen C , Andrew G , Beaulieu C . Extensive deep gray matter volume reductions in children and adolescents with fetal alcohol spectrum disorders. Alcohol Clin Exp Res. 2011;35(8):1404–17. 21575012 10.1111/j.1530-0277.2011.01476.x

[9] Willoughby KA , Sheard ED , Nash K , Rovet J . Effects of prenatal alcohol exposure on hippocampal volume, verbal learning, and verbal and spatial recall in late childhood. J Int Neuropsychol Soc. 2008;14(6):1022–33. 18954482 10.1017/S1355617708081368

[10] Anacker C , Hen R . Adult hippocampal neurogenesis and cognitive flexibility - linking memory and mood. Nat Rev Neurosci. 2017;18(6):335–46. 28469276 10.1038/nrn.2017.45 PMC6261347

[11] Clelland CD , Choi M , Romberg C , Clemenson GD Jr , Fragniere A , Tyers P et al. A functional role for adult hippocampal neurogenesis in spatial pattern separation. Science. 2009;325(5937):210–3. 19590004 10.1126/science.1173215 PMC2997634

[12] Danielson NB , Kaifosh P , Zaremba JD , Lovett-Barron M , Tsai J , Denny CA , et al. Distinct Contribution of Adult-Born Hippocampal Granule Cells to Context Encoding. Neuron. 2016;90(1):101–12. 26971949 10.1016/j.neuron.2016.02.019 PMC4962695

[13] Denny CA , Burghardt NS , Schachter DM , Hen R , Drew MR . 4- to 6-week-old adult-born hippocampal neurons influence novelty-evoked exploration and contextual fear conditioning. Hippocampus. 2012;22(5):1188–201. 21739523 10.1002/hipo.20964 PMC3193906

[14] Nakashiba T , Cushman JD , Pelkey KA , Renaudineau S , Buhl DL , McHugh TJ , et al. Young dentate granule cells mediate pattern separation, whereas old granule cells facilitate pattern completion. Cell. 2012;149(1):188–201. 22365813 10.1016/j.cell.2012.01.046 PMC3319279

[15] Swan AA , Clutton JE , Chary PK , Cook SG , Liu GG , Drew MR . Characterization of the role of adult neurogenesis in touch-screen discrimination learning. Hippocampus. 2014;24(12):1581–91. 25074617 10.1002/hipo.22337 PMC4236255

[16] Hellemans KG , Sliwowska JH , Verma P , Weinberg J . Prenatal alcohol exposure: fetal programming and later life vulnerability to stress, depression and anxiety disorders. Neurosci Biobehav Rev. 2010;34(6):791–807. 19545588 10.1016/j.neubiorev.2009.06.004 PMC5518679

[17] O’Connor MJ , Paley B . Psychiatric conditions associated with prenatal alcohol exposure. Dev Disabil Res Rev. 2009;15(3):225–34. 19731386 10.1002/ddrr.74

[18] Pei J , Denys K , Hughes J , Rasmussen C . Mental health issues in fetal alcohol spectrum disorder. J Ment Health. 2011;20(5):438–48. 10.3109/09638237.2011.577113 21780939

[19] Streissguth AP , O’Malley K . Neuropsychiatric implications and long-term consequences of fetal alcohol spectrum disorders. Seminars in Clinical Neuropsychiatry. 2000;5(3):177–90. 11291013 10.1053/scnp.2000.6729

[20] Choi IY , Allan AM , Cunningham LA . Moderate fetal alcohol exposure impairs the neurogenic response to an enriched environment in adult mice. Alcohol Clin Exp Res. 2005;29(11):2053–62. 16340464 10.1097/01.alc.0000187037.02670.59

[21] Cunningham LA , Newville J , Li L , Tapia P , Allan AM , Valenzuela CF . Prenatal Alcohol Exposure Leads to Enhanced Serine 9 Phosphorylation of Glycogen Synthase Kinase-3beta (GSK-3beta) in the Hippocampal Dentate Gyrus of Adult Mouse. Alcohol Clin Exp Res. 2017;41(11):1907–16. 28865114 10.1111/acer.13489 PMC5659904

[22] Kajimoto K , Allan A , Cunningham LA . Fate analysis of adult hippocampal progenitors in a murine model of fetal alcohol spectrum disorder (FASD). PLoS ONE. 2013;8(9):e73788. 24040071 10.1371/journal.pone.0073788 PMC3770701

[23] Clemenson GD , Henningfield CM , Stark CEL . Improving Hippocampal Memory Through the Experience of a Rich Minecraft Environment. Front Behav Neurosci. 2019;13:57. 30949036 10.3389/fnbeh.2019.00057 PMC6437107

[24] Clemenson GD , Lee SW , Deng W , Barrera VR , Iwamoto KS , Fanselow MS , et al. Enrichment rescues contextual discrimination deficit associated with immediate shock. Hippocampus. 2015;25(3):385–92. 25330953 10.1002/hipo.22380 PMC4398310

[25] Ethen MK , Ramadhani TA , Scheuerle AE , Canfield MA , Wyszynski DF , Druschel CM , et al. Alcohol consumption by women before and during pregnancy. Maternal and Child Health Journal. 2009;13(2):274–85. 18317893 10.1007/s10995-008-0328-2 PMC6090563

[26] Lagace DC , Whitman MC , Noonan MA , Ables JL , DeCarolis NA , Arguello AA , et al. Dynamic contribution of nestin-expressing stem cells to adult neurogenesis. J Neurosci. 2007;27(46):12623–9. 18003841 10.1523/JNEUROSCI.3812-07.2007 PMC3718551

[27] Madisen L , Zwingman TA , Sunkin SM , Oh SW , Zariwala HA , Gu H , et al. A robust and high-throughput Cre reporting and characterization system for the whole mouse brain. Nature Neuroscience. 2010;13(1):133–40. 20023653 10.1038/nn.2467 PMC2840225

[28] Gustus K , Lozano E , Newville J , Li L , Valenzuela CF , Cunningham LA . Resistance of Postnatal Hippocampal Neurogenesis to Alcohol Toxicity in a Third Trimester-Equivalent Mouse Model of Gestational Alcohol Exposure. Alcohol Clin Exp Res. 2019. 10.1111/acer.14207 PMC6904425 31573091

[29] Brady ML , Allan AM , Caldwell KK . A limited access mouse model of prenatal alcohol exposure that produces long-lasting deficits in hippocampal-dependent learning and memory. Alcoholism, Clinical and Experimental Research. 2012;36(3):457–66. 10.1111/j.1530-0277.2011.01644.x PMC3572784 21933200

[30] Carrica L , Li L , Newville J , Kenton J , Gustus K , Brigman J , Cunningham LA . Genetic inactivation of hypoxia inducible factor 1-alpha (HIF-1alpha) in adult hippocampal progenitors impairs neurogenesis and pattern discrimination learning. Neurobiology of Learning and Memory. 2018;157:79–85. 30521851 10.1016/j.nlm.2018.12.002 PMC6389421

[31] Gustus KC , Li L , Chander P , Weick JP , Wilson MC , Cunningham LA . Genetic inactivation of synaptosomal-associated protein 25 (SNAP-25) in adult hippocampal neural progenitors impairs pattern discrimination learning but not survival or structural maturation of newborn dentate granule cells. Hippocampus. 2018;28(10):735–44. 29995325 10.1002/hipo.23008 PMC6467575

[32] Sahay A , Scobie KN , Hill AS , O’Carroll CM , Kheirbek MA , Burghardt NS , et al. Increasing adult hippocampal neurogenesis is sufficient to improve pattern separation. Nature. 2011;472(7344):466–70. 21460835 10.1038/nature09817 PMC3084370

[33] Huckleberry KA , Shue F , Copeland T , Chitwood RA , Yin W , Drew MR . Dorsal and ventral hippocampal adult-born neurons contribute to context fear memory. Neuropsychopharmacology. 2018. 10.1038/s41386-018-0109-6 PMC6180107 29941977

[34] Goodlett CR , Peterson SD . Sex differences in vulnerability to developmental spatial learning deficits induced by limited binge alcohol exposure in neonatal rats. Neurobiol Learn Mem. 1995;64(3):265–75. 8564380 10.1006/nlme.1995.0009

[35] Sharrett-Field L , Butler TR , Reynolds AR , Berry JN , Prendergast MA . Sex differences in neuroadaptation to alcohol and withdrawal neurotoxicity. Pflugers Arch. 2013;465(5):643–54. 23559099 10.1007/s00424-013-1266-4 PMC3654022

[36] McHugh TJ , Jones MW , Quinn JJ , Balthasar N , Coppari R , Elmquist JK , et al. Dentate gyrus NMDA receptors mediate rapid pattern separation in the hippocampal network. Science. 2007;317(5834):94–9. 17556551 10.1126/science.1140263

[37] Kheirbek MA , Klemenhagen KC , Sahay A , Hen R . Neurogenesis and generalization: a new approach to stratify and treat anxiety disorders. Nature Neuroscience. 2012;15(12):1613–20. 23187693 10.1038/nn.3262 PMC3638121

[38] Kheirbek MA , Tannenholz L , Hen R . NR2B-dependent plasticity of adult-born granule cells is necessary for context discrimination. J Neurosci. 2012;32(25):8696–702. 22723709 10.1523/JNEUROSCI.1692-12.2012 PMC3388607

[39] Tronel S , Belnoue L , Grosjean N , Revest JM , Piazza PV , Koehl M , et al. Adult-born neurons are necessary for extended contextual discrimination. Hippocampus. 2012;22(2):292–8. 21049483 10.1002/hipo.20895

[40] Drew LJ , Kheirbek MA , Luna VM , Denny CA , Cloidt MA , Wu MV , et al. Activation of local inhibitory circuits in the dentate gyrus by adult-born neurons. Hippocampus. 2016;26(6):763–78. 26662922 10.1002/hipo.22557 PMC4867135

[41] Luna VM , Anacker C , Burghardt NS , Khandaker H , Andreu V , Millette A , et al. Adult-born hippocampal neurons bidirectionally modulate entorhinal inputs into the dentate gyrus. Science. 2019;364(6440):578–83. 31073064 10.1126/science.aat8789 PMC6800071

[42] Bergami M , Masserdotti G , Temprana SG , Motori E , Eriksson TM , Gobel J , et al. A critical period for experience-dependent remodeling of adult-born neuron connectivity. Neuron. 2015;85(4):710–7. 25661179 10.1016/j.neuron.2015.01.001

[43] Lemaire V , Tronel S , Montaron MF , Fabre A , Dugast E , Abrous DN . Long-lasting plasticity of hippocampal adult-born neurons. The Journal of Neuroscience: The Official Journal of the Society for Neuroscience. 2012;32(9):3101–8. 22378883 10.1523/JNEUROSCI.4731-11.2012 PMC6622037

[44] Pallas-Bazarra N , Jurado-Arjona J , Navarrete M , Esteban JA , Hernandez F , Avila J , et al. Novel function of Tau in regulating the effects of external stimuli on adult hippocampal neurogenesis. EMBO J. 2016;35(13):1417–36. 27198172 10.15252/embj.201593518 PMC4876034

[45] Trinchero MF , Buttner KA , Sulkes Cuevas JN , Temprana SG , Fontanet PA , Monzon-Salinas MC , et al. High Plasticity of New Granule Cells in the Aging Hippocampus. Cell Rep. 2017;21(5):1129–39. 29091753 10.1016/j.celrep.2017.09.064

[46] Trinchero MF , Herrero M , Monzon-Salinas MC , Schinder AF . Experience-Dependent Structural Plasticity of Adult-Born Neurons in the Aging Hippocampus. Front Neurosci. 2019;13:739. 31379489 10.3389/fnins.2019.00739 PMC6651579

[47] Tronel S , Fabre A , Charrier V , Oliet SH , Gage FH , Abrous DN . Spatial learning sculpts the dendritic arbor of adult-born hippocampal neurons. Proceedings of the National Academy of Sciences of the United States of America. 2010;107(17):7963–8. 20375283 10.1073/pnas.0914613107 PMC2867872

[48] Zhao C , Jou J , Wolff LJ , Sun H , Gage FH . Spine morphogenesis in newborn granule cells is differentially regulated in the outer and middle molecular layers. The Journal of Comparative Neurology. 2015;523(10):1588. 26109421 10.1002/cne.23800

[49] Drew MR , Huckleberry KA . Modulation of Aversive Memory by Adult Hippocampal Neurogenesis. Neurotherapeutics. 2017;14(3):646–61. 28488160 10.1007/s13311-017-0528-9 PMC5509626

[50] Deng W , Mayford M , Gage FH . Selection of distinct populations of dentate granule cells in response to inputs as a mechanism for pattern separation in mice. Elife. 2013;2:e00312. 23538967 10.7554/eLife.00312 PMC3602954

[51] Chancey JH , Adlaf EW , Sapp MC , Pugh PC , Wadiche JI , Overstreet-Wadiche LS . GABA depolarization is required for experience-dependent synapse unsilencing in adult-born neurons. J Neurosci. 2013;33(15):6614–22. 23575858 10.1523/JNEUROSCI.0781-13.2013 PMC3657840

[52] Ge S , Yang CH , Hsu KS , Ming GL , Song H . A critical period for enhanced synaptic plasticity in newly generated neurons of the adult brain. Neuron. 2007;54(4):559–66. 17521569 10.1016/j.neuron.2007.05.002 PMC2040308

[53] Gu Y , Arruda-Carvalho M , Wang J , Janoschka SR , Josselyn SA , Frankland PW , et al. Optical controlling reveals time-dependent roles for adult-born dentate granule cells. Nature Neuroscience. 2012;15(12):1700–6. 23143513 10.1038/nn.3260 PMC3509272

[54] Marin-Burgin A , Mongiat LA , Pardi MB , Schinder AF . Unique processing during a period of high excitation/inhibition balance in adult-born neurons. Science. 2012;335(6073):1238–42. 22282476 10.1126/science.1214956 PMC3385415

[55] Schmidt-Hieber C , Jonas P , Bischofberger J . Enhanced synaptic plasticity in newly generated granule cells of the adult hippocampus. Nature. 2004;429(6988):184–7. 15107864 10.1038/nature02553

[56] Snyder JS , Cameron HA . Could adult hippocampal neurogenesis be relevant for human behavior? Behavioural Brain Research. 2012;227(2):384–90. 21736900 10.1016/j.bbr.2011.06.024 PMC3210392

[57] Dieni CV , Nietz AK , Panichi R , Wadiche JI , Overstreet-Wadiche L . Distinct determinants of sparse activation during granule cell maturation. J Neurosci. 2013;33(49):19131–42. 24305810 10.1523/JNEUROSCI.2289-13.2013 PMC3850038

[58] Mongiat LA , Esposito MS , Lombardi G , Schinder AF . Reliable activation of immature neurons in the adult hippocampus. PLoS One. 2009;4(4):e5320. 19399173 10.1371/journal.pone.0005320 PMC2670498

[59] Alvarez DD , Giacomini D , Yang SM , Trinchero MF , Temprana SG , Büttner KA , et al. A disynaptic feedback network activated by experience promotes the integration of new granule cells. Science. 2016;354(6311):459–65. 27789840 10.1126/science.aaf2156

[60] Jessberger S , Clark RE , Broadbent NJ , Clemenson GD Jr , Consiglio A , Lie DC et al. Dentate gyrus-specific knockdown of adult neurogenesis impairs spatial and object recognition memory in adult rats. Learn Mem. 2009;16(2):147–54. 19181621 10.1101/lm.1172609 PMC2661246

[61] Suarez-Pereira I , Carrion AM . Updating stored memory requires adult hippocampal neurogenesis. Sci Rep. 2015;5:13993. 26358557 10.1038/srep13993 PMC4566137

[62] Witter MP . The perforant path: projections from the entorhinal cortex to the dentate gyrus. Prog Brain Res. 2007;163:43–61. 17765711 10.1016/S0079-6123(07)63003-9

[63] Vivar C , Potter MC , Choi J , Lee JY , Stringer TP , Callaway EM , et al. Monosynaptic inputs to new neurons in the dentate gyrus. Nature Communications. 2012;3:1107. 10.1038/ncomms2101 PMC4603575 23033083

[64] Woods NI , Vaaga CE , Chatzi C , Adelson JD , Collie MF , Perederiy JV , et al. Preferential Targeting of Lateral Entorhinal Inputs onto Newly Integrated Granule Cells. J Neurosci. 2018;38(26):5843–53. 29793975 10.1523/JNEUROSCI.1737-17.2018 PMC6021988

[65] Erwin SR , Sun W , Copeland M , Lindo S , Spruston N , Cembrowski MS . A Sparse, Spatially Biased Subtype of Mature Granule Cell Dominates Recruitment in Hippocampal-Associated Behaviors. Cell Rep. 2020;31(4):107551. 32348756 10.1016/j.celrep.2020.107551

[66] van de Weerd HA . Evaluation of environmental enrichment for laboratory mice. Vet Q. 1997;19(sup1):59. 22047439 10.1080/01652176.1997.9694816

[67] van de Weerd HA , Baumans V , Koolhaas JM , van Zutphen LF . Strain specific behavioural response to environmental enrichment in the mouse. J Exp Anim Sci. 1994;36(4-5):117–27. 7948063

[68] Kajimoto K , Valenzuela CF , Allan AM , Ge S , Gu Y , Cunningham LA . Prenatal alcohol exposure alters synaptic activity of adult hippocampal dentate granule cells under conditions of enriched environment. Hippocampus. 2016;26(8):1078–87. 27009742 10.1002/hipo.22588 PMC4949153

[69] Kasai H , Matsuzaki M , Noguchi J , Yasumatsu N , Nakahara H . Structure-stability-function relationships of dendritic spines. Trends Neurosci. 2003;26(7):360–8. 12850432 10.1016/S0166-2236(03)00162-0

[70] Chidambaram SB , Rathipriya AG , Bolla SR , Bhat A , Ray B , Mahalakshmi AM , et al. Dendritic spines: Revisiting the physiological role. Prog Neuropsychopharmacol Biol Psychiatry. 2019;92:161–93. 30654089 10.1016/j.pnpbp.2019.01.005

[71] Jungenitz T , Beining M , Radic T , Deller T , Cuntz H , Jedlicka P , et al. Structural homo- and heterosynaptic plasticity in mature and adult newborn rat hippocampal granule cells. Proc Natl Acad Sci U S A. 2018;115(20):E4670–E9. 29712871 10.1073/pnas.1801889115 PMC5960324

[72] Bergami M , Berninger B . A fight for survival: the challenges faced by a newborn neuron integrating in the adult hippocampus. Developmental Neurobiology. 2012;72(7):1016–31. 22488787 10.1002/dneu.22025

[73] Deisseroth K , Singla S , Toda H , Monje M , Palmer TD , Malenka RC . Excitation-neurogenesis coupling in adult neural stem/progenitor cells. Neuron. 2004;42(4):535–52. 15157417 10.1016/s0896-6273(04)00266-1

[74] Song J , Zhong C , Bonaguidi MA , Sun GJ , Hsu D , Gu Y , et al. Neuronal circuitry mechanism regulating adult quiescent neural stem-cell fate decision. Nature. 2012;489(7414):150–4. 22842902 10.1038/nature11306 PMC3438284

[75] Kirschen GW , Shen J , Tian M , Schroeder B , Wang J , Man G , et al. Active Dentate Granule Cells Encode Experience to Promote the Addition of Adult-Born Hippocampal Neurons. J Neurosci. 2017;37(18):4661–78. 28373391 10.1523/JNEUROSCI.3417-16.2017 PMC5426562

[76] Brady ML , Diaz MR , Iuso A , Everett JC , Valenzuela CF , Caldwell KK . Moderate prenatal alcohol exposure reduces plasticity and alters NMDA receptor subunit composition in the dentate gyrus. The Journal of Neuroscience: the Official Journal of the Society for Neuroscience. 2013;33(3):1062–7. 23325244 10.1523/JNEUROSCI.1217-12.2013 PMC3563269

[77] Helfer JL , White ER , Christie BR . Enhanced deficits in long-term potentiation in the adult dentate gyrus with 2nd trimester ethanol consumption. PloS One. 2012;7(12):e51344. 23227262 10.1371/journal.pone.0051344 PMC3515437

[78] Sutherland RJ , McDonald RJ , Savage DD . Prenatal exposure to moderate levels of ethanol can have long-lasting effects on hippocampal synaptic plasticity in adult offspring. Hippocampus. 1997;7(2):232–8. 9136052 10.1002/(SICI)1098-1063(1997)7:2<232::AID-HIPO9>3.0.CO;2-O

[79] Varaschin RK , Akers KG , Rosenberg MJ , Hamilton DA , Savage DD . Effects of the cognition-enhancing agent ABT-239 on fetal ethanol-induced deficits in dentate gyrus synaptic plasticity. The Journal of Pharmacology and Experimental Therapeutics. 2010;334(1):191–8. 20308329 10.1124/jpet.109.165027 PMC2912053

[80] Samudio-Ruiz SL , Allan AM , Sheema S , Caldwell KK . Hippocampal N-methyl-D-aspartate receptor subunit expression profiles in a mouse model of prenatal alcohol exposure. Alcoholism, Clinical and Experimental Research. 2010;34(2):342–53. 10.1111/j.1530-0277.2009.01096.x PMC3600588 19951292

[81] Hamilton GF , Jablonski SA , Schiffino FL , St Cyr SA , Stanton ME , Klintsova AY . Exercise and environment as an intervention for neonatal alcohol effects on hippocampal adult neurogenesis and learning. Neuroscience. 2014;265:274–90. 24513389 10.1016/j.neuroscience.2014.01.061 PMC4005875

[82] Ieraci A , Herrera DG . Single alcohol exposure in early life damages hippocampal stem/progenitor cells and reduces adult neurogenesis. Neurobiology of Disease. 2007;26(3):597–605. 17490887 10.1016/j.nbd.2007.02.011

[83] Redila VA , Olson AK , Swann SE , Mohades G , Webber AJ , Weinberg J , et al. Hippocampal cell proliferation is reduced following prenatal ethanol exposure but can be rescued with voluntary exercise. Hippocampus. 2006;16(3):305–11. 16425237 10.1002/hipo.20164

[84] Boldrini M , Fulmore CA , Tartt AN , Simeon LR , Pavlova I , Poposka V , et al. Human Hippocampal Neurogenesis Persists throughout Aging. Cell Stem Cell. 2018;22(4):589–99 e5. 29625071 10.1016/j.stem.2018.03.015 PMC5957089

[85] Eriksson PS , Perfilieva E , Björk-Eriksson T , Alborn A-M , Nordborg C , Peterson DA , et al. Neurogenesis in the adult human hippocampus. Nature Medicine. 1998;4(11). 10.1038/3305 9809557

[86] Manganas LN , Zhang X , Li Y , Hazel RD , Smith SD , Wagshul ME , et al. Magnetic resonance spectroscopy identifies neural progenitor cells in the live human brain. Science. 2007;318(5852):980–5. 17991865 10.1126/science.1147851 PMC4039561

[87] Spalding KL , Bergmann O , Alkass K , Bernard S , Salehpour M , Huttner HB , et al. Dynamics of hippocampal neurogenesis in adult humans. Cell. 2013;153(6):1219–27. 23746839 10.1016/j.cell.2013.05.002 PMC4394608

[88] Sorrells SF , Paredes MF , Cebrian-Silla A , Sandoval K , Qi D , Kelley KW , et al. Human hippocampal neurogenesis drops sharply in children to undetectable levels in adults. Nature. 2018. 10.1038/nature25975 PMC6179355 29513649

[89] Chokroborty-Hoque A , Alberry B , Singh SM . Exploring the complexity of intellectual disability in fetal alcohol spectrum disorders. Front Pediatr. 2014;2:90. 25207264 10.3389/fped.2014.00090 PMC4143882

[90] Benjamin LE , Keshet E . Conditional switching of vascular endothelial growth factor (VEGF) expression in tumors: induction of endothelial cell shedding and regression of hemangioblastoma-like vessels by VEGF withdrawal. Proc Natl Acad Sci U S A. 1997;94(16):8761–6. 9238051 10.1073/pnas.94.16.8761 PMC23118

[91] Dodge NC , Thomas KGF , Meintjes EM , Molteno CD , Jacobson JL , Jacobson SW . Reduced Hippocampal Volumes Partially Mediate Effects of Prenatal Alcohol Exposure on Spatial Navigation on a Virtual Water Maze Task in Children. Alcohol Clin Exp Res. 2020;44(4):844–55. 32196695 10.1111/acer.14310 PMC7166198

[92] Riikonen R , Salonen I , Partanen K , Verho S . Brain perfusion SPECT and MRI in foetal alcohol syndrome. Developmental Medicine and Child Neurology. 1999;41(10):652–9. 10587040 10.1017/s0012162299001358

[93] Sowell ER , Lu LH , O’Hare ED , McCourt ST , Mattson SN , O’Connor MJ , et al. Functional magnetic resonance imaging of verbal learning in children with heavy prenatal alcohol exposure. Neuroreport. 2007;18(7):635–9. 17426589 10.1097/WNR.0b013e3280bad8dc

[94] Uban KA , Herting MM , Wozniak JR , Sowell ER , Cifasd . Sex differences in associations between white matter microstructure and gonadal hormones in children and adolescents with prenatal alcohol exposure. Psychoneuroendocrinology. 2017;83:111–21. 28609669 10.1016/j.psyneuen.2017.05.019 PMC5877456

[95] Weinberg J , Sliwowska JH , Lan N , Hellemans KG . Prenatal alcohol exposure: foetal programming, the hypothalamic-pituitary-adrenal axis and sex differences in outcome. J Neuroendocrinol. 2008;20(4):470–88. 18266938 10.1111/j.1365-2826.2008.01669.x PMC8942074

[96] Yagi S , Galea LAM . Sex differences in hippocampal cognition and neurogenesis. Neuropsychopharmacology. 2019;44(1):200–13. 30214058 10.1038/s41386-018-0208-4 PMC6235970

